# Importance of muscle strength to maintain mobility, but not to maintain postural balance in older women: Cross-sectional study

**DOI:** 10.1016/j.clinsp.2024.100504

**Published:** 2024-09-24

**Authors:** Roberta Alexandra Gonçalves de Toledo Evangelista, Alexandre Lopes Evangelista, Rita de Cássia Ernandes, Guilherme Carlos Brech, Reinaldo Nonato da Silva, Matheus Henrique dos Santos Lino, Danilo Sales Bocalini, Myriam de Graaf, Luis Mochizuki, Jose Maria Soares-Junior, Edmund Chada Baracat, Júlia Maria D'Andréa Greve, Luiz Eugênio Garcez-Leme, Angelica Castilho Alonso

**Affiliations:** a Program in Aging Science, Universidade São Judas Tadeu (USJT), São Paulo, SP, Brazil; b Laboratory for the Study of Movement, Institute of Orthopedics and Traumatology, Faculdade de Medicine, Universidade de São Paulo (USP), São Paulo, SP, Brazil; c Laboratories of Exercise Physiology and Experimental Physiology, Biochemistry of Physical Education and Sport Center at Universidade Federal do Espírito Santo, Vitoria, ES, Brazil; d Institute of Sport and Exercise Sciences, Universität Münster: Westfalische Wilhelms-Universitat Munster, Germany; e School of Arts, Sciences, and Humanities, Universidade de São Paulo, São Paulo, SP, Brazil; f Disciplina de Ginecologia, Departamento de Obstetrícia e Ginecologia, Hospital das Clínicas, Faculdade de Medicina da Universidade de São Paulo (FMUSP), São Paulo, SP, Brazil

**Keywords:** Older adults, Muscular strength, Postural Balance and mobility

## Abstract

**Background:**

Acknowledging the relationship between postural control and muscle strength in lower limbs is important to find persons with high fall risk and to design fall prevention exercise programs.

**Objective:**

To investigate the connection between knee muscle strength, semi-static postural balance, and functional mobility in irregularly active older women.

**Methods:**

One hundred and ten older women aged 60 to 85 years underwent a semi-static postural balance test in a force and functional mobility measured by Timed Up & Go (TUG) with and without Cognitive Tasks (CT). Muscle strength of the knee was assessed by isokinetic dynamometry at 60°/s.

**Results:**

A negative correlation between the functional mobility (TUG with and without CT) and the muscular strength of the knee was observed. Regarding semi-static balance, there was a low negative correlation with peak torque corrected for body weight of the extensors with the following variables with eyes open: Mean Velocity Dominant limb (D) (*r* = -0.19; p = 0.03); and anteroposterior range Non-Dominant limb (ND) (r = -0.19; p = 0.04); with eyes closed: mediolateral range ND limb (*r* = -0.21; p = 0.02) and Mean Velocity ND limb (*r* = -0.18; p = 0.05).

**Conclusions:**

This study found that better functional mobility was associated with greater muscle strength in the knee in elderly women. There were weak negative correlations between knee extensor strength and some measures of semi-static postural balance, suggesting that different motor control actions are required to maintain semi-static balance and mobility. In the case of semi-static balance, a lower level of muscle response is required than in functional mobility.

## Highlights

• Motor control actions are required to maintain semi-static balance and mobility.
• Quadriceps and hamstring muscles are important for mobility.
• Quadriceps and hamstring muscles are not important for semi-static balance.

## Introduction

Aging is a process of morphological, physiological, biochemical, and psychological changes that result in a slow performance of the organic system and as a consequence, it reduces functional capacity.^1^ Disregarding muscular, neurological, and environmental changes,^2^ a person loses about 10% to 15% of muscle strength per decade from the fifties onward, associated with a reduction in motor unit recruitment speed. This loss of strength tends to reach 30% after the age of 70^3^ and increases the risk of falls, thereby enhancing the risk of fracture and often leading to disabilities and dependencies.^4^

Falls are the main cause of healthcare problems in older adults. Between 28% and 35% of older adults experience falls of any type and this number tends to increase in the oldest population.^5^^,^^6^ The risk of falls among older adults involves several elements, such as changes in lower limb muscle strength and postural balance.^7^^,^^8^ Deficits in these systems have been identified as intrinsic risk factors for injuries at different ages and they have been shown to increase with age.^9^ Maintaining postural balance requires sensorimotor detection of body position and movement by integrating this information in the Central Nervous System (CNS), besides appropriate execution of musculoskeletal responses controlling and coordinating the entire kinetic chain.^10^ Body position in relation to space is determined by integrating sensorimotor, vestibular, and visual information. As visual acuity decreases over the years, the ability to detect spatial information diminishes as well.^7^^,^^11^ These functional modifications in the elderly result in a greater vulnerability in postural balance that can lead to falls.^7^

Experimental studies applied resistance exercise to improve postural balance. Kendrick et al.^12^ and Kumar et al.^13^ have conducted systematic reviews of clinical trials with different types of exercise, including resistance exercise, to improve balance and decrease the fear of falling, and pointed out that interventions reduced the fear of falling to a weak to moderate degree only immediately after the intervention. However, the high risk of bias in most of the included studies suggests that findings should be interpreted with caution.

Muscle strength and postural balance are physical skills that use independent neuromuscular components, and they complete each other so that a person can accomplish their daily life and sports routines. A possible interrelation between these two components is not clear yet. Some studies^14^, ^15^, ^16^, ^17^ could not discover a relation between strength and semi-static postural balance, while others^18^^,^^19^ showed a positive correlation. The different assessment techniques, ages, and groups usually from both genders are likely responsible for these differing findings.

Given this context, this work is justified by the need to better understand the interrelationship between muscle strength and postural balance in the elderly, aiming to develop more effective interventions to reduce the risk of falls and improve the quality of life for this population.

Thus, the aim of this study was to correlate knee muscle strength with semi-static postural balance and mobility in irregularly active older women.

## Material and methods

### Experimental design, local and ethics

This was a cross-sectional study, performed at the Universidade São Judas Tadeu (USJT/SP) together with the Motion Study Laboratory of the Institute of Orthopedics and Traumatology, Hospital das Clínicas, Faculty of Medicine, Universidade de São Paulo. All participants gave their written informed consent before participating in this study, which was approved by the Ethics Committee at the Faculty of Medicine, University of São Paulo (registration number 723/2009).

### Subjects

In this study, a convince sample with a total of 110 irregularly active older women aged 60 to 85 took part in this study. Inclusion of participants was based on the following criteria: absence of vestibular, proprioceptive, auditory, neurological and/or mental system impairment assessed by Mini Mental State Examination (MESM); no complaints regarding dizziness or loss of balance; no history of any great injury in the lower limbs in the past six months; no lower limb or trunk procedures that could influence postural balance; no great joint movement limitations in the ankle, knee and/or hip; presenting normal gait, without limping. Before inclusion, all participants were evaluated by a geriatrician. If a participant did not perform any of the postural balance or muscle strength tests they were excluded from the study.

### Assessment

Participants were evaluated and screened in the Motion Study Laboratory of the Institute of Orthopedics and Traumatology, Hospital das Clinics, University of São Paulo School of Medicine. All volunteers filled in a questionnaire with demographic and anthropometric data prior to the assessment. The participants’ level of physical activity was assessed with the International Physical Activity Questionnaire (IPAQ), a short version, adapted to the Brazilian population. The IPAQ classifies exercise into five different categories (namely: very active; active; irregularly active A; irregularly active B, and sedentary), based on the frequency and duration of moderate to vigorous physical activity in the preceding week.^20^

The semi-static postural balance assessment (posturography) was performed on a standard force platform (AccuSway plus, Advanced Mechanical Technology Inc AMTI), which was set up for a 100 Hz sampling frequency with a 10 Hz cutoff frequency fourth-order Butterworth filter. In the standing position, the ground reaction forces, and the body sway of each volunteer were recorded. Marks for each foot were made on a sheet of paper fixed to the platform at 4 points (hallux, head of the fifth metatarsal, lateral and medial malleolus); then, the support base was registered by applying 10 pounds to each of the marked points. The volunteers remained on the platform in bipedal support, heels apart at the marks on the sheet of paper, arms resting along the length of the body, head still, and eyes fixed on a point one meter away, 10 cm below the participants’ height. A total of three sessions (60s each) of each condition with eyes open and closed were performed in bipedal support, with a one-minute rest interval in between. By the end of each procedure, the data were saved, and the platform was calibrated. The arithmetic mean of the outcome parameters over all three tests in each condition was calculated and automatically processed by the *Balance Clinic* software analysis system The measured parameters were the Center of Pressure (COP), Force (F), and Moments (M) in two directions (medio-lateral-X and antero-posterior-Y). COP positions were determined using the data obtained in the Antero-Posterior (AP) and Medio-Lateral (ML) directions. The analyzed variables were: displacement amplitudes in the medio-lateral plane (sum X maximum + minimum X) and antero-posterior (sum Y maximum + minimum Y); average speed (total distance traveled by the COP divided by the collection time), measured in cm/s and elliptical area of 95% of displacement of the COP.^8^

A functional mobility assessment was performed using the TUG with and without Cognitive Task (TUG-CT), which is a test where the individual starts in a sitting position, stands up, walks for three meters at their usual speed, returns, and sits back down.^21^ In the TUG-CT, the procedure was like the traditional TUG, except the participant was required to say the names of animals out loud, as previously described.^22^^,^^23^

An isokinetic evaluation of the knee flexors and extensors was performed as described in a previous study^24^ using the Biodex® multi-joint System3 isokinetic dynamometer (Biodex Medical Systems Inc. Shirley, NY, USA). The knee extension and flexion were tested in concentric/concentric mode, starting with the dominant lower limb. The volunteers were positioned with the lateral condyle of the femur aligned with the dynamometer mechanical axis. The range of motion was set starting at 90° flexion and reaching 20° extension, with correction of the force of gravity at the halfway point. Two sets of five uninterrupted repetitions of consecutive knee extension and flexion were performed, with an interval of 60s in each set. The procedure was repeated for the non-dominant limb. For data analysis, the values of the second set were used, due to the effects of motor learning seen in consecutive sets of the same test on the isokinetic dynamometer.^25^ The angular velocity was set to 60°/s and the recorded outcomes were the peak torque corrected for body mass (PT//BW; in %) and the total work (TW; in J).

### Statistical analysis

The data were stored and analyzed using SPSS 20.0 for Windows (SPSS, Inc.). The descriptive analysis was done by means of an average, standard deviation, median, minimum and maximum. The Kolmogorov-Smirnov test was performed to verify whether the continuous variables had a normal distribution. Spearman's correlation test was used to correlate the numerical variables. For the entire sample, p < 0.05 was considered significant.

## Results

Of the 110 participants, 23 (20.9%) were classified as irregularly active A and 87 (79.1%) as irregularly active B according to the IPAQ. None of them met the criteria of frequency duration and intensity of exercise. Age, anthropometric characteristics, and the results of the strength and postural balance tests are described in Table 1.

**Table 1:** Age, anthropometric variables, muscle strength and postural balance of the elderly women.

	Mean (SD)	Minimum	Maximum
Age (years)	67.4 (5.9)	60	85
Weight (kg)	71.0 (12.8)	36	112
Height (cm)	1.56 (.06)	1.36	1.68
BMI (cm2)	29.1 (4.8)	18.37	46.62
**Isokinetic dynamometry**			
*Dominant extensor*			
PT/BW (%)	123.1 (33.6)	11	214.1
Total Work (J)	295.4 (90.6)	99	673.5
*Dominant flexor*			
PT/BW (%)	55.7 (17.8)	13.1	129.7
Total Work (J)	139.3 (44.4)	23.1	254
*Non Dominant extensor*			
PT/BW (%)	125.7 (29.7)	57.4	201
Total Work (J)	301.4 (80.8)	104.4	599.2
*Non dominant flexor*			
PT/BW (%)	57.6 (18.0)	19.6	111.2
Total Work (J)	144.3 (47.5)	44.9	286.7
**Functional Mobility**			
TUGT(s)	9.9 (2.5)	5	18.5
Cognitive TUGT (s)	11.8 (3.0)	5.8	20.2
**Postural Balance – Force Platform**			
*Eyes Open*			
Range displacement ML(cm)	1.2 (0.5)	0.38	3.22
Range displacement AP (cm)	2.2 (0.7)	0.66	4.08
Mean Velocity (°/sec)	0.87 (0.2)	0.38	2.04
Area 95% (cm^2^)	1.8 (1.4)	0.14	8.47
*Eyes Closed*			
Range displacement ML (cm)	1.1 (0.7)	-1.47	3.01
Range displacement AP (cm)	2.2(1.4)	-2.79	5.42
Mean Velocity (°/sec)	1.03 (0.5)	-2.26	2.05
Area 95% (cm^2^)	2.0 (1.5)	0.1	8.29

SD, Standard Deviation; BMI, Body Mass Index; PT/BW, Peak Torque corrected for Body Weight; TUGT, Timed Up & Go Test; ML, Mediolatral; AP, Anterioposterior.

There was a weak negative correlation between functional mobility TUG with and without a cognitive task and the knee flexors and extensors muscle strength (Table 2).

**Table 2:** Correlation between functional mobility and muscle strength of knee extensors and flexors.

	PT/BW	Total Work	PT/BW	Total Work
	r (p)		r (p)	
	**Dominant extensor**		**Dominant flexor**	
**TUGT (sec)**	-.27 (0.001)*	-.35 (0.001)*	-.16 (.08)	-.34 (0.001)*
**TUGT Cognitive (sec)**	-.26 (.01)*	-.35 (0.001)*	-.18(.07)	-.37(0.001)*
	**Non Dominant extensor**		**Non Dominant flexor**	
**TUGT (sec)**	.19 (.03)*	-,32 (0,001)*	-.19 (.04)*	-30 (0.001)*
**TUGT Cognitive (sec)**	-.20 (0.5)*	-,32 (0.001)*	-,17 (.09)	-.30 (0.001)*

r, Spearman Correlation.

^a^ p ≤ 0.05.

PT/BW, Peak Torque corrected for Body Weight; TUGT, Timed Up & Go Test.

There was only a weak negative correlation between mean velocity with the PT/BW dominant extensors. With eyes open and PT/BW non-dominant extensor with antero-posterior range and with eyes closed with medio-lateral range (Table 3).

**Table 3:** Correlation between semi-static postural balance and muscle strength of knee extensors and flexors.

	PT/BW	Total Work	PT/BW	Total Work
	r (p)		r (p)	
**Eyes open**	**Dominant extensor**		**Dominant flexor**	
Range ML (cm)	-.07 (0.42)	.06 (0.47)	-.12 (0.19)	-.00 (0.96)
Range AP (cm)	-.07 (0.41)	.12(.18)	-.15 (0.09)	.06 (0.47)
Mean Velocity (°/sec)	-.19 (0.03)*	-.09 (0.30)	-.12 (0.18)	-.04 (0.61)
Area 95% (cm^2^)	-.08 (0.35)	.11 (0.25)	-.15 (0.09)	.03 (0.73)
**Eyes Closed**				
Range ML (cm)	-.11 (0.25)	-.03 (0.73)	-.13 (0.16)	-.04 (0.62)
Range AP (cm)	-.04 (0.68)	.05 (0.56)	-.11 (0.23)	.02 (0.81)
Mean Velocity (°/sec)	-.17 (0.06)	-.01 (0.85)	-.16 (0.07)	-.01 (0.85)
Area 95% (cm^2^)	-.11 (0.25)	.07 (0.46)	-.16 (0.08)	.02 (0.78)
**Eyes Open**	**Non Dominant extensor**		**Non Dominant flexor**	
Range ML (cm)	-.12 (0.18)	.03 (0.74)	-.14 (0.11)	-.04 (0.61)
Range AP (cm)	-.19 (0.04)*	.07 (0.45)	-.17 (0.06)	.02 (0.81)
Mean Velocity (°/sec)	-.12 (0.18)	-.03 (0.73)	-.16 (0.09)	-.10 (0.26)
Area 95% (cm^2^)	-.15 (0.10)	-.08 (0.39)	-.17 (0.06)	-.01 (0.87)
**Eyes Closed**				
Range ML (cm)	-.21 (0.02)*	-.07 (0.46)	-.12 (0.17)	-.04 (0.66)
Range AP (cm)	-.16 (0.08)	.02 (0.83)	-.10 (0.27)	.02 (0.77)
Mean Velocity (°/sec)	-.18 (0.05)*	-.02 (0.82)	-.15 (0.09)	-.05 (0.59)
Area 95% (cm^2^)	-.14 (0.12)	-.06 (0.52)	-.11 (0.25)	.04 (0.61)

r, Spearman Correlation.

^a^ p ≤ 0.05.

PT/BW, Peak Torque corrected for Body Weight; ML, Mediolateral; AP, Anterioposterior.

## Discussion

The present analysis reveals the low to non-existent linear correlation between functional mobility and semi-static balance, respectively, with the parameters the knee flexion and extension torque and TUG. Other studies^16^, ^17^, ^18^ also found low to no correlation between the semi-static balance and functional mobility in older adults,^26^ young people^27^ and adults.^28^ One explanation is that quasi-static balance and functional mobility demand different motor control actions. In particular, in the semi-static condition, disturbances demand a lower level of muscle response^29^^,^^30^ as the mechanical conformation of the joints and the mechanical properties of the ligaments, tendons, muscles, and cartilage, such as stiffness, viscosity, tension versus speed, tension versus muscle-tendon length relationship, also contribute to joint position maintenance in an important way. So, in static balance, the demand for torque is lower compared to when joints are moving. Therefore, the performance of maximum muscle strength must bear little relation to the performance in static balance.^10^

The older adult's performance in keeping a standing upright posture does not depend on the maximum torque of the knee flexor muscles. The isokinetic test's largest indicators for knee flexion and the parameters of postural balance in semi-static posture are not linearly correlated; this lack of correlation can be explained by neurophysiological mechanisms (motor pathway activation). The regulation of postural balance and force production requires different spinal activations by the supra-spinal centers. Beck et al.^31^ investigated the excitability of lower motor neurons during the execution of isometric ankle strength training and balance training, in conditions of instability, and showed that there is different activation. On the other hand, Morita et al.^32^ showed the cortical activation patterns during the execution of a maximum strength task are facilitated by the execution of isometric plantar flexion and dorsiflexion, and that they were unchanged when a static balance task was performed. In maintaining static balance, the importance of sensory information is to monitor the position of the body and adjust the level of joint stiffness to ensure stability.^10^

There was a low linear correlation between the performance of both conventional and cognitive TUG with peak torque and knee extension/flexion work, which corroborates with Serra et al.^8^ who state that peak knee torque for movements in the sagittal plane is associated more with dynamic rather than static tests; dynamic conditions demand greater reflex responses of the muscles of the lower limbs and activation of tonic and phasic muscles to successfully stabilize the center of gravity on the support base. This is the case in the TUG test, which requires the individual to stand up from a chair, walk three meters, return, and sit down again.^33^ During the test, which is associated with fall risk, the action of the knee flexors and extensors is highlighted.^34^ In the initial contact of the gait cycle, there is a quadriceps concentric work extending the knee; in the load response phase, 18° knee flexion occurs to mitigate the effect of fast weight transfer. During medium and terminal support (30%‒50% gait cycle), as the body rolls forward on the forefoot, the knee completes its extension. When the swing phase begins, the knee is at 60° flexion, then it returns to a 30° and resumes the knee extension cycle.^35^ Likewise, the sit-to-stand movement features concentric and eccentric quadriceps action to control the displacement of the center of gravity and to keep it within the base of support depending on the quadriceps' muscular strength.^8^

The limitations of this study are that it only evaluated elderly women, which may restrict the generalization of the results to other populations. Furthermore, the low correlation found between muscular strength and postural balance suggests that other factors may influence these capabilities. Testing was performed in a controlled laboratory environment, which may not reflect actual conditions. Additionally, the study did not consider the influence of sensory factors and individual adaptation capacity.

The clinical implications of this study are associated with the demands of different assessments, planning and adequate development of training programs with respect to the specificities of each type of physical fitness. Another important aspect is that most falls occur in dynamic conditions, such as walking, or going up and down steps, and injury/fall risk assessments should, therefore, be performed under dynamic conditions to accurately identify potential balance problems. The postural balance and muscle strength of the lower limbs must be tested, compared, and trained in a complementary way throughout life, as the biological aging process of the neuromuscular system has a deleterious effect on both balance components and muscle strength.^26^, ^28^

## Conclusion

This study found that better functional mobility was associated with greater muscle strength in the knee extensors and flexors in elderly women. There were weak negative correlations between knee extensor strength and some measures of semi-static postural balance, and no correlation was found with knee flexors, suggesting that different motor control actions are required to maintain semi-static balance and mobility. In the case of semi-static balance, a lower level of muscle response is required than in functional mobility.

## Authors’ contributions

Roberta Alexandra Gonçalves de Toledo Evangelista: Literature review; data collection; writing of the manuscript.

Alexandre Lopes Evangelista: Research concept and study design; data analysis and interpretation.

Rita de Cássia Ernandes: Literature review; data collection.

Guilherme Carlos Brech: Research concept and study design; writing of the manuscript.

Reinaldo Nonato da Silva: Literature review; data collection.

Matheus Henrique dos Santos Lino: Literature review; data collection.

Danilo Sales Bocalini: Research concept and study design reviewing/editing a draft of the manuscript.

Myriam de Graaf: Data analysis and interpretation; reviewing/editing a draft of the manuscript.

Luis Mochizuki: Research concept and study design; data analysis and interpretation.

Jose Maria Soares-Junior: Supporting Investigation and writing-review & editing.

Edmund Chada Baracat: Supporting Investigation and writing-review & editing.

Júlia Maria D'Andréa Greve: Research concept and study design; reviewing/editing a draft of the manuscript.

Luiz Eugênio Garcez-Leme: Research concept and study design; reviewing/editing a draft of the manuscript.

Angelica Castilho Alonso: Research concept and study design; statistical analyses; writing of the manuscript.

## Conflicts of interest

The authors declare no conflicts of interest.
